# Sex differences in diffusion-weighted imaging outcomes in autosomal dominant Alzheimer’s disease

**DOI:** 10.1093/braincomms/fcag081

**Published:** 2026-03-12

**Authors:** Averi Giudicessi, Elouise Koops, Ana Baena, Sergio Alvarez, Monica Vidal, Lusiana Martinez, Nikole A Bonillas Félix, Isabela Gonzalez, Randy Medrano, Catarina Tristão-Pereira, Vincent Malotaux, Bing He, Clara Vila-Castelar, Heidi I L Jacobs, David Aguillon, Alice Cronin-Golomb, Yakeel T Quiroz

**Affiliations:** Department of Psychiatry, Massachusetts General Hospital, Harvard Medical School, Boston, MA 02129, USA; Department of Psychological and Brain Sciences, Boston University, Boston, MA 02215, USA; Department of Radiology, Massachusetts General Hospital, Harvard Medical School, Boston, MA 02129, USA; Grupo de Neurociencias de Antioquia, Universidad de Antioquia, Medellín 050001, Colombia; Department of Radiology, Hospital Pablo Tobon Uribe, Medellín 050001, Colombia; Department of Radiology, Hospital Pablo Tobon Uribe, Medellín 050001, Colombia; Department of Psychiatry, Massachusetts General Hospital, Harvard Medical School, Boston, MA 02129, USA; Department of Psychological and Brain Sciences, Boston University, Boston, MA 02215, USA; Department of Psychiatry, Massachusetts General Hospital, Harvard Medical School, Boston, MA 02129, USA; Department of Psychiatry, Massachusetts General Hospital, Harvard Medical School, Boston, MA 02129, USA; Department of Psychological and Brain Sciences, Boston University, Boston, MA 02215, USA; Department of Psychiatry, Massachusetts General Hospital, Harvard Medical School, Boston, MA 02129, USA; Department of Psychiatry, Massachusetts General Hospital, Harvard Medical School, Boston, MA 02129, USA; Department of Psychological and Brain Sciences, Boston University, Boston, MA 02215, USA; Department of Psychiatry, Massachusetts General Hospital, Harvard Medical School, Boston, MA 02129, USA; Department of Psychological and Brain Sciences, Boston University, Boston, MA 02215, USA; Department of Psychiatry, Massachusetts General Hospital, Harvard Medical School, Boston, MA 02129, USA; Department of Psychological and Brain Sciences, Boston University, Boston, MA 02215, USA; Department of Psychiatry, Massachusetts General Hospital, Harvard Medical School, Boston, MA 02129, USA; Department of Radiology, Massachusetts General Hospital, Harvard Medical School, Boston, MA 02129, USA; Grupo de Neurociencias de Antioquia, Universidad de Antioquia, Medellín 050001, Colombia; Department of Psychological and Brain Sciences, Boston University, Boston, MA 02215, USA; Department of Psychiatry, Massachusetts General Hospital, Harvard Medical School, Boston, MA 02129, USA; Department of Psychological and Brain Sciences, Boston University, Boston, MA 02215, USA; Grupo de Neurociencias de Antioquia, Universidad de Antioquia, Medellín 050001, Colombia

**Keywords:** Alzheimer’s disease, sex differences, white matter microstructure, diffusion tensor imaging, autosomal dominant Alzheimer’s disease

## Abstract

Alzheimer's disease is characterized by amyloid-β and tau protein accumulation. Growing evidence suggests that white matter degeneration contributes to disease progression. Despite Alzheimer's disease being more prevalent in women, understanding of sex differences in white matter microstructure across the Alzheimer's disease continuum remains limited. This study investigated sex-specific patterns of white matter integrity in individuals genetically predisposed to autosomal dominant Alzheimer's disease. We analysed data from 63 individuals (30 presenilin-1 glutamic acid to Alanine at codon 280 (*PSEN1* E280A) mutation carriers, 33 non-carriers) from a Colombian kindred with early-onset autosomal dominant Alzheimer's disease. Participants underwent diffusion-weighted imaging, amyloid and tau positron emission tomography and cognitive assessment. Using fixel-based analysis, we examined fibre density, fibre-bundle cross-section and combined fibre density and cross-section across major white matter tracts. Linear regression models assessed sex differences in white matter microstructure and examined how sex moderated relations between pathological burden, white matter integrity and cognitive performance. Females showed trends towards higher fibre cross-section than males in the anterior thalamic radiation (β = 0.057, *P* = 0.004), forceps minor of the corpus callosum (β = 0.047, *P* = 0.012) and inferior fronto-occipital fasciculus (β = 0.030, *P* = 0.015), but lower values in the cingulum (cingulate gyrus portion) (β = −0.07, *P* = 0.031). Sex appeared to moderate the association between pathology and white matter in multiple tracts. However, these findings did not survive correction for multiple comparisons and should be interpreted as exploratory. In the right temporal superior longitudinal fasciculus, females showed relatively preserved integrity compared to males as tau burden increased (β = 0.23, *P* = 0.016), while males exhibited greater amyloid-β-associated disruption than females in the uncinate fasciculus (β = −0.14, *P* = 0.05). Sex also moderated relations between white matter integrity and cognition, with males showing stronger structure–function coupling than females in tracts such as the cingulum (hippocampus portion), forceps major and corticospinal tract. Our findings revealed significant sex-specific patterns of white matter microstructural alterations in autosomal dominant Alzheimer's disease, with females showing preserved fibre cross-section in key tracts and slower rates of white matter deterioration with increasing tau pathology in memory-related circuits, whereas males demonstrated advantages in structure–function coupling. These data highlight the importance of considering sex differences in understanding white matter alterations in Alzheimer's disease and suggest the potential utility of considering sex differences in the development of personalized interventions and clinical trials for persons with Alzheimer's disease.

## Teaser

Giudicessi *et al*. report that females with inherited early-onset Alzheimer's disease carrying the presenilin-1 mutation show preserved connections between brain regions and slower decline as tau protein accumulates, whereas males show stronger dependence on these connections to support memory. These patterns suggest complementary sex-specific protective mechanisms in Alzheimer's disease.

## Graphical Abstract

**Graphical Abstract:**
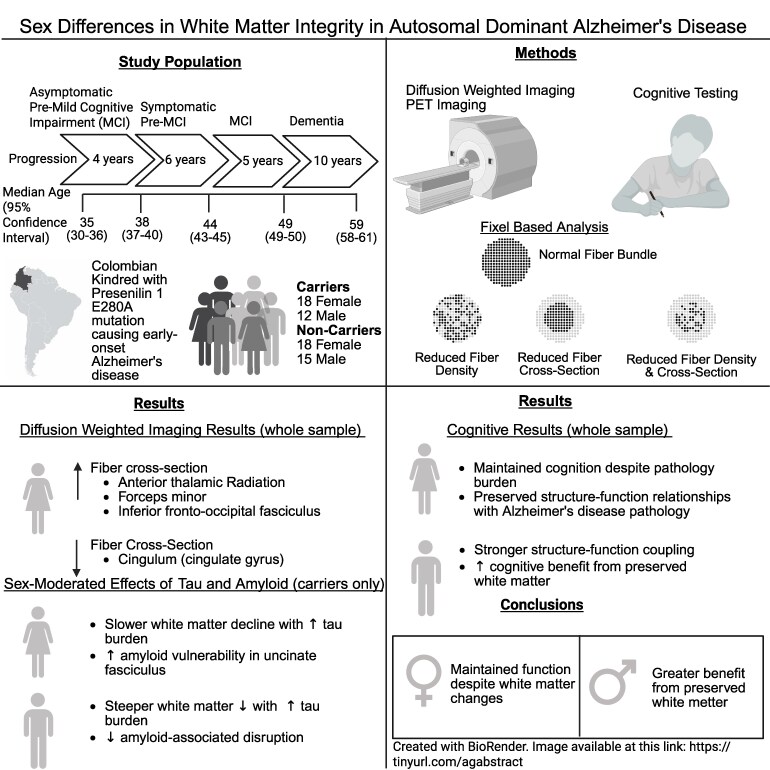

## Introduction

Alzheimer’s disease (Ad) stands as the leading cause of dementia among older adults, distinguished by the presence of amyloid-β (Aβ) and hyperphosphorylated tau proteins.^1^ Though predominantly associated with grey matter pathology, white matter degeneration is also a common structural alteration in individuals with AD.^2,3^ These changes in white matter microstructure often result from cerebrovascular disease and significantly contribute to Ad-related neurodegeneration.^4^ Assessing these alterations in vivo is feasible through diffusion-weighted imaging (DWI), which examines fibre tracts based on the restriction of motion of water molecules, and can noninvasively examine neural microtissue organization.^5^

Sex differences in ageing and Ad have garnered considerable attention in research. Emerging evidence suggests disparities in white matter abnormalities during pathological ageing^6,7^ with females tending to exhibit higher white matter hyperintensity load than males, even after accounting for midlife vascular factors.^8^ This suggests that sex-specific factors may contribute to the development of these pathologies.^9-12^ Despite Ad being more prevalent in women, understanding sex differences in white matter alterations in the context of Ad pathology remains inconclusive.

Research indicates notable microstructural differences in white matter fibre tracts among individuals with mild cognitive impairment (MCI) and symptomatic Ad, compared to cognitively healthy individuals, particularly in areas such as the forceps major and forceps minor of the corpus callosum, inferior fronto-occipital fasciculus, cingulum-angular bundles and posterior cingulum.^13,14^ These findings point to white matter damage as a significant aspect of Ad pathology, distinct from grey matter deterioration and often occurring independently.^13,15^ Further, these white matter alterations have been associated with cognitive performance, with specific tracts relating to memory function, including the uncinate fasciculus for verbal memory and posterior corpus callosum fibres for memory and executive function.^16-18^ While sex differences have been extensively studied in grey matter volume changes and various white matter pathologies,^7,19,20^ research on sex differences in white matter microstructure using DWI methods is scarce. Recent work using free-water corrected diffusion tensor imaging (DTI) (a specific DWI method) has demonstrated sex-specific patterns in Ad, with males showing higher-free water index values than females in the right anterior thalamic radiation, right corticospinal tract, right superior longitudinal fasciculus and right cerebral peduncle.^12^ Differences in fractional anisotropy were primarily observed in the left anterior thalamic radiation and right corticospinal tract, suggesting differential white matter tract disorganization between males and females. Studies comparing young healthy adults and those with MCI have indicated that males tend to display more microstructural damage than females^21^ highlighting the need for further sex-specific research in white matter integrity across the Ad continuum.

Given the findings of sex-specific white matter alterations in Ad, their functional implications are important to understand. Sex differences may extend beyond simple vulnerability patterns to include differential mechanisms of cognitive resilience, whereby males and females may employ distinct neural compensation strategies when facing pathological burden.^22,23^ This cognitive resilience, the ability to maintain function despite underlying disease processes, may be differentially supported by white matter networks in each sex.^24,25^ Sex-specific compensation mechanisms could explain why identical levels of pathological burden may result in different cognitive outcomes between males and females, and potentially why certain white matter tracts may be preferentially affected in one sex over another.

The objective of this study was to investigate sex-specific differences in white matter microstructure in a homogenous cohort, using fixel-based analysis (FBA), which enables fibre-specific assessment of white matter microstructure and morphology. The cohort comprised carriers of the presenilin 1 (*PSEN1)* E280A mutation, a cause of autosomal dominant Ad (ADAD) and non-carriers of the mutation. Carriers of the *PSEN1* E280A mutation develop mild cognitive impairment at a median age of 44 years and dementia at 49 years,^26^ providing the ability to examine preclinical pathology in adults who are genetically determined to develop Ad dementia with high certainty. We hypothesized that there would be sex-specific patterns of white matter vulnerability, with differential baseline white matter integrity between males and females. Further, we hypothesized that sex would moderate the impact of *PSEN1* mutation status on white matter microstructure. Among mutation carriers, where pathological burden is elevated and variable, we hypothesized that sex would moderate the relationships between pathological burden (amyloid and tau) and white matter integrity, with differential patterns of vulnerability between male and female carriers. Finally, we hypothesized that sex would moderate structure–function relationships, reflecting differential cognitive resilience mechanisms between males and females. The goal of the study was to provide insights into disease progression and ultimately to facilitate the identification of targeted, sex-specific therapeutic interventions. Given that this cohort carries a single autosomal-dominant mutation, we acknowledge that findings should be interpreted cautiously with respect to generalizability to sporadic Ad, though considerable pathological and clinical overlap exists between autosomal dominant and sporadic forms of Ad.

## Materials and methods

### Participants

The study sample included a total of 63 individuals, comprising 33 non-carriers and 30 mutation carriers (23 unimpaired carriers and 7 cognitively impaired carriers). Participants were enrolled in this study between September 2011 and March 2024 from the Alzheimer’s Prevention Initiative registry, a registry including members of a large Colombian kindred with the *PSEN1* E280A mutation leading to early-onset ADAD. Participants travelled from Colombia to Boston to complete study procedures. Participants included in the present study ranged in age from 20 to 55 years. Sex was defined as sex assigned at birth and was recorded as male or female for all participants. The Functional Assessment Staging Tool was used to measure clinical impairment in participants, with scores range from 1 (normal) to 7 (severe dementia) with a score ≤ 2 indicating no objective impairment.^27^ Participants and the study staff were blind to the individuals’ *PSEN1* genetic status. Participants underwent a MRI scan, Pittsburgh Compound B (PiB) positron emission tomography (PET) for *in vivo* quantification of Aβ burden, 18F-flortaucipir (FTP) PET for quantification of tau burden and a neurological and neuropsychological evaluation. All participants provided informed consent. Study procedures were carried out in accordance with ethical standards from the Helsinki Declaration and were approved by the Bioethics Committee of the University of Antioquia (Medellin, Colombia) and the Institutional Review Board of the Massachusetts General Hospital (USA). Researchers and participants were blind to genetic status.

## Data acquisition

### Clinical and cognitive assessment

Global cognitive functioning was assessed with the Mini-Mental State Examination (MMSE; range 0–30).^28^ Episodic verbal memory function was assessed using the Spanish-Colombian version of the Consortium to Establish a Registry for Alzheimer’s Disease (CERAD; range 0–10)^29^ Word List Learning delayed recall subtest, in which participants are asked to recall as many words as they can remember from a prior learned list of ten words.

### Imaging acquisition and protocol

Participants completed an MRI and PET scan at Massachusetts General Hospital. The MRI protocol included diffusion-weighted images using a single shot spin echo planar imaging sequence [repetition time (TR) = 8040 msec, echo time (TE) = 84 msec, flip angle (FA) = 80°, field of view = 256 × 256 mm, voxel size = 2.0 × 2.0 × 2.0 mm, 30 diffusion-sensitizing gradients with a b-value of 700 s/mm^2^] and 5 non-diffusion-weighted images (b = 0 s/mm^2^). T1 magnetization–prepared rapid-acquisition gradient-echo sequence (MPRAGE) [TR = 2300 ms, TE = 2.95 ms, FA = 9°, resolution = 1.1 × 1.1 × 1.2 mm] was also acquired for each subject and was used to compute intracranial volume using FreeSurfer.

PET data were acquired on a Siemens ECAT HR + scanner (3D mode; 63 image planes; 15.2 cm axial field of view; 5.6 mm transaxial resolution; 2.4 mm slice interval). Tau data were acquired using the 18F-flortaucipir (FTP) tracer from 80–100 min post-injection in 4 × 5-min frames. Aβ data were acquired using the 11C-Pittsburgh compound B (PiB) tracer using a 60-min dynamic protocol and analysed by the Logan reference method.^30^ Outcome measures were standardized uptake value ratio (SUVr) for FTP and distribution volume ratio (DVR) for PiB, using cerebellar grey matter as the reference region for both outcomes. Co-registration between PET and T1 images was performed using affine transformation, and all PET data were sampled using FreeSurfer-derived regions of interest (ROIs). A composite region comprising frontal, lateral temporal, parietal and retrosplenial cortices was used to represent PiB DVR as a measure of global amyloid burden.^31^ Tau burden was assessed in three regions of interest known to show early tau accumulation in Ad: entorhinal cortex, inferior temporal cortex and precuneus. These regions were selected based on established patterns of tau progression in Ad pathology, corresponding to early and intermediate Braak stages. Because we had no hypotheses about lateralization, left and right hemispheres were averaged for each FTP ROI.

### Image preprocessing

Preprocessing of diffusion-weighted images was completed using MRTrix3 (www.mrtrix.org) and included denoising of data (dwidenoise),^32^ motion and distortion correction (dwifslpreproc),^33^ bias field correction (dwibiascorrect)^34^ and up-sampling DWI spatial resolution by a factor of 2 in all three dimensions using cubic b-spline interpolation (mrgrid), to an isotropic voxel size of 1.25 mm.^35^ Skull stripping involved a multi-step procedure. Initially, brain extraction was performed on the B0 images using dwi2mask; subsequently, this brain extraction was linearly co-registered to the MNI 1 mm B0 image using the FMRIB linear image registration tool with an affine model. Finally, the co-registration matrix was inverted and applied to the MNI 1 mm mask to obtain the brain mask for each native B0 image and thus the final B0 brain extractions. Intensity normalization across participants was performed by deriving scale factors from the median intensity in select voxels of white matter, grey matter and cerebrospinal fluid (CSF) in b = 0 s/mm^2^ images, then applying these across each subject image using mtnormalise.

### Fibre orientation distribution estimation

Following preprocessing, fibre orientation distribution (FODs) were computed using Single-Shell, 3-Tissue Constrained Spherical Deconvolution (SS3T-CSD), with group averaged response functions for white matter, grey matter and CSF.^36,37^ Response functions for the three tissue types were estimated using the dwi2response function.

Spatial correspondence was achieved by first generating a group-specific population template with an iterative registration and averaging approach^30^ using FOD images from all participants. Each participant's FOD image was then registered to the template via a FOD-guided non-linear registration.^30,35^ To ensure consistency across participants, a template mask was computed by warping all participant masks into template space and determining the intersection.

### Fixel-based metrics

We employed FBA rather than standard DTI because DTI cannot resolve multiple fibre orientations within a single voxel and produces voxel-averaged metrics that lack fibre specificity in crossing fibre regions. In contrast, FBA provides fibre-specific quantification by separately measuring microstructural and macrostructural properties of individual fibre populations within each voxel. Each such fibre population within a voxel is termed a ‘fixel’. We derived metrics of apparent fibre density (FD), fibre-bundle cross-section (log-FC) and a combined measure of fibre density and cross-section (FDC) for each subject across white matter fixels. These metrics provide complementary biological information: FD reflects the density of the axons within a fibre bundle (similar to intra-axonal volume), log-FC captures macrostructural changes in the physical size of the fibre bundle (similar to tract atrophy or expansion in the cross-section area), and FDC combines both microstructural density and macrostructural size to provide an integrated measure of overall tract integrity. More detailed methodology for FBA along with interpretations for fixel-based metrics have been described by Raffelt *et al*. (2017).

### Fibre density

We used the Apparent Fibre Density framework to compute a measure related to the intra-axonal restricted compartment at each fixel.^35^ According to this framework, a quantitative measure of fibre density was derived from FOD images, given that the integral of the FOD along a particular direction is proportional to the intra-axonal volume of axons aligned in that direction. The fibre density measure is therefore specifically sensitive to alterations at a microstructural, within-voxel level. The fibre density value for each fixel in each participant was obtained by estimating each fixel's contribution to the total DWI signal in a voxel using SS3T-CSD.^36^ Following spatial normalization, the fibre density value for each fixel in each subject was then assigned to a fixel template mask.

### Fibre bundle cross-section

While fibre density enables estimation of differences in intra-axonal volume of a fibre pathway within a voxel, another likely scenario is that a loss of axons results in atrophy of a fibre bundle across its entire cross-section. The fibre bundle cross-section metric is computed to be sensitive to such a change, as described by Raffelt *et al*. (2017). Morphological differences in the fixel cross-section (in the plane perpendicular to the fixel direction) were estimated for each fixel by using the non-linear warps to compute the change in fibre bundle cross-section required to spatially normalize the subject image to the template image. With respect to the template frame of reference, fibre bundle cross-section values >1 indicate a larger fibre cross-section in the subject, while fibre bundle cross-section values <1 indicate a smaller cross-section.^31^

### Fibre density and cross-section

Because group differences may manifest as changes to both within-voxel fibre density and macrostructural changes in fibre-bundle cross-section, we additionally computed a metric that combined both sources of information, namely the combined measure of FDC.^31^

### Tract of interest analysis

We performed tract of interest analyses to investigate potential degeneration of selective fibre pathways using the Johns Hopkins University (JHU) White-Matter Tractography Atlas (https://identifiers.org/neurovault.collection:264). This approach reduces multiple comparison burden compared to whole-brain fixel-wise analysis while maintaining comprehensive anatomical coverage. Specifically, we used the JHU-ICBM-tracts-maxprob-thr25-1 mm atlas, which includes 20 major white matter tracts: bilateral anterior thalamic radiations, corticospinal tracts, cingulum (cingulate gyrus portion), cingulum (hippocampal portion), inferior fronto-occipital fasciculi, inferior longitudinal fasciculi, superior longitudinal fasciculi, superior longitudinal fasciculi (temporal part), uncinate fasciculi, as well as the forceps major and forceps minor. Mean values for FD, FC and the combined FDC metrics were computed across the fixels in each defined tract and compared across groups.

### Statistical analyses

Statistical analyses were performed using R statistical software (version 4.4.2). Prior to analysis, data were examined for normality and transformed when necessary. *PSEN1* E280A mutation carriers from the Colombian kindred constitute a highly specific and well-characterized population, and our analyses were designed to maximize information from this cohort (*N* = 63). Effect sizes and confidence intervals are reported alongside significance tests to provide meaningful clinical insights regardless of statistical significance. Demographic characteristics were compared across groups using Kruskal–Wallis (ANOVA) for continuous variables and Fisher’s exact test for categorical variables.

Our primary analysis used linear regression models to examine sex differences in white matter microstructure and how sex moderates the relation between *PSEN1* E280A mutation status, pathology and cognitive performance.

### Sex differences in white matter microstructure

We assessed sex differences in white matter microstructure by modelling each diffusion metric as the dependent variable with sex as the primary predictor while controlling for *PSEN1* E280A status, age and intracranial volume.

### Sex *PSEN1* status interactions

We tested for the interaction effect between *PSEN1* E280A status and sex to determine if the impact of the mutation on white matter microstructure differed between males and females, while controlling for age and intracranial volume.

### Sex-moderated pathology-white matter relations

We examined how white matter microstructure related to pathological markers, specifically tau PET uptake in three regions (entorhinal, inferior temporal and precuneus cortices) and global Aβ burden. Among *PSEN1* E280A carriers specifically, we examined whether sex moderated the relation between pathology and white matter measures (White matter ∼ Pathology × Sex). These carrier-specific models included interactions between pathological measures and sex, while controlling for age and intracranial volume, to identify potential sex-specific vulnerability patterns within carriers.

### Sex-moderated cognition-white matter relations

We investigated associations between white matter microstructure and cognitive performance by assessing whether the diffusion-derived microstructural and integrity measures predicted cognitive functioning differently by sex. We constructed general linear models with cognitive measures (MMSE and CERAD word list delayed recall) as outcome variables and diffusion metrics as predictors. Each model included the diffusion measure of interest, sex and the interaction between sex and the diffusion measure (sex × diffusion measure) as predictors of interest, while controlling for *PSEN1* E280A status, age and intracranial volume. Models were analysed across the whole sample to identify sex-moderated relations with cognition. Models were analysed across the whole sample to identify sex-moderated relations with cognition.

In all models, *PSEN1* E280A status was coded as carrier/non-carrier, sex as male/female and intracranial volume was included to control for head size variation. For normally distributed measures, standard linear regression was used, while robust regression with MM-estimation was employed for non-normally distributed measures to minimize the influence of outliers and heteroscedasticity. No participants were excluded based on outlier detection, as cognitive and pathological variability across preclinical to symptomatic disease stages represents clinically meaningful variation in this cohort (see Supplementary Table S1 for detailed outlier assessment). All analyses acknowledge the limited statistical power inherent in studies of rare genetic mutations, with emphasis placed on effect size estimation and clinical relevance rather than solely on statistical significance.

## Results

### Participant characteristics

A summary of demographic, clinical and biomarker characteristics across groups (female carriers, female non-carriers, male carriers, male non-carriers) is presented in Table 1. Significant age differences were observed across groups (*P* = 0.007) with female carriers being the oldest (40.5 ± 5.8 years) and male non-carriers being the youngest (33.7 ± 3.5 years). Education levels did not differ significantly between groups (*P* = 0.13). Carriers demonstrated significantly worse cognitive performance than non-carriers on both cognitive measures. MMSE scores were lowest in female carriers (27 ± 4) compared to both non-carrier females (29 ± 1) and non-carrier males (29 ± 1) and male carriers (29 ± 1; *P* = 0.008). CERAD delayed recall showed similar patterns, with both carrier groups (female: 5 ± 3; male: 5 ± 3) performing more poorly than their respective non-carrier counterparts (female: 8 ± 1; male: 7 ± 1, *P* = 0.002). As expected, carriers exhibited significantly elevated Ad pathology burden across all biomarkers relative to non-carriers. Global Aβ load was markedly higher in both female carriers (1.82 ± 0.43) and male carriers (1.53 ± 0.22) than in non-carriers (1.12 ± 0.05 and 1.11 ± 0.04, *P* < 0.001). Tau burden followed a similar pattern across all regions, with female carriers showing the highest levels in entorhinal cortex (1.91 ± 0.68), followed by male carriers (1.50 ± 0.64), while in both non-carrier groups, it was low (female: 1.07 ± 0.24; male: 1.04 ± 0.19, *P* < 0.001). This pattern was consistent across inferior temporal cortex and precuneus regions (all *P* ≤ 0.002).

**Table 1:** Demographics by group

	Female carrier, *N* = 18^a^	Female non-carrier, *N* = 18^a^	Male carrier, *N* = 12^a^	Male non-carrier, *N* = 15^a^	*P*-value^b^
Age (years)	40.5 (5.8)	36.7 (4.9)	36.5 (5.8)	33.7 (3.5)	0.007
Education (years)	10 (5)	12 (4)	9 (4)	12 (4)	0.13
MMSE score	27 (4)	29 (1)	28 (2)	29 (1)	0.008
Word List Delayed Recall	5 (3)	8 (1)	5 (3)	7 (1)	0.002
FAST score					<0.001
1	7 (39%)	17 (94%)	9 (75%)	15 (100%)	
2	5 (28%)	1 (5.6%)	2 (17%)	0 (0%)	
3	5 (28%)	0 (0%)	1 (8.3%)	0 (0%)	
4	1 (5.6%)	0 (0%)	0 (0%)	0 (0%)	
TAU SUVR Inferior Temporal	1.66 (0.71)	1.19 (0.11)	1.29 (0.21)	1.17 (0.12)	0.002
TAU SUVR Entorhinal	1.91 (0.68)	1.07 (0.24)	1.50 (0.64)	1.04 (0.19)	<0.001
TAU SUVR Precuneus	2.23 (1.45)	1.09 (0.15)	1.28 (0.43)	1.07 (0.10)	<0.001
Global Amyloid (PIB DVR)	1.82 (0.43)	1.12 (0.05)	1.53 (0.22)	1.11 (0.04)	<0.001

^a^Mean (SD); *n* (%).

^b^Kruskal–Wallis rank sum test; Fisher's exact test.

### Sex differences in white matter microstructure

**Figure 1:**
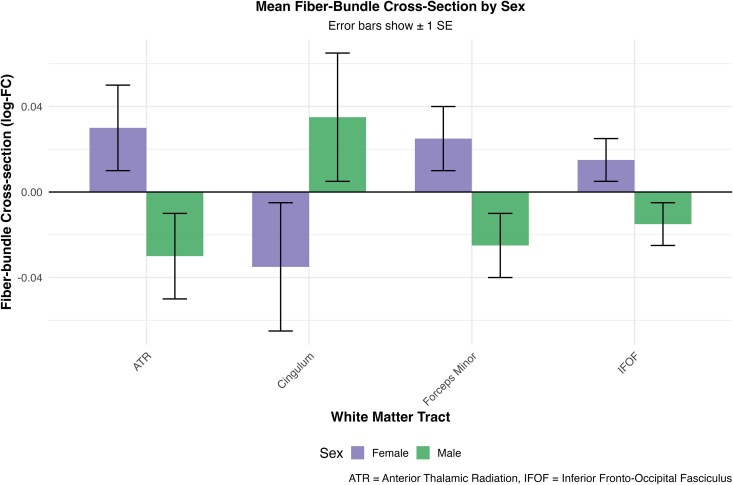
**Sex differences in mean fibre-bundle cross-section across white matter tracts.** Bar plot comparing log-transformed fibre cross-section (log-FC) between males (*n* = 27) and females (*n* = 36) across four major white matter tracts, analysed using linear regression controlling for *PSEN1* E280A status, age and intracranial volume. Statistical analysis: Linear regression models controlling for *PSEN1* E280A status, age and intracranial volume. Females show significantly higher fibre cross-section in the anterior thalamic radiation (β = 0.06, *t* = 2.99, *P* = 0.004), forceps minor (β = 0.05, *t* = 2.58, *P* = 0.012) and inferior fronto-occipital fasciculus (β = 0.03, *t* = 2.50, *P* = 0.015). Males demonstrate higher values in the cingulum (cingulate gyrus portion) (β = −0.07, *t* = −2.21, *P* = 0.031). Error bars represent ±1 standard error. Each bar represents one measurement per participant.

Regression analyses controlling for *PSEN1* E280A status, age and intracranial volume revealed significant sex differences across multiple white matter tracts. Females showed significantly higher fibre-bundle cross-section (log-FC) values than males in the anterior thalamic radiation (β = 0.06, 95% CI [0.02–0.01] *P* = 0.004), forceps minor (β = 0.05, 95% CI [0.007–0.057], *P* = 0.012) and inferior fronto-occipital fasciculus (β = 0.030, 95% CI [0.007, 0.054], *P* = 0.015). Females showed lower fibre-bundle cross-section (log-FC) values in the cingulum (cingulate gyrus portion) (β = −0.07, 95% CI [−0.132, −0.008], *P* = 0.031) (Fig. 1). After FDR correction, none of these sex differences remained statistically significant (*P*_FDR < 0.05).

### Sex × *PSEN1* E280A status interactions

**Figure 2:**
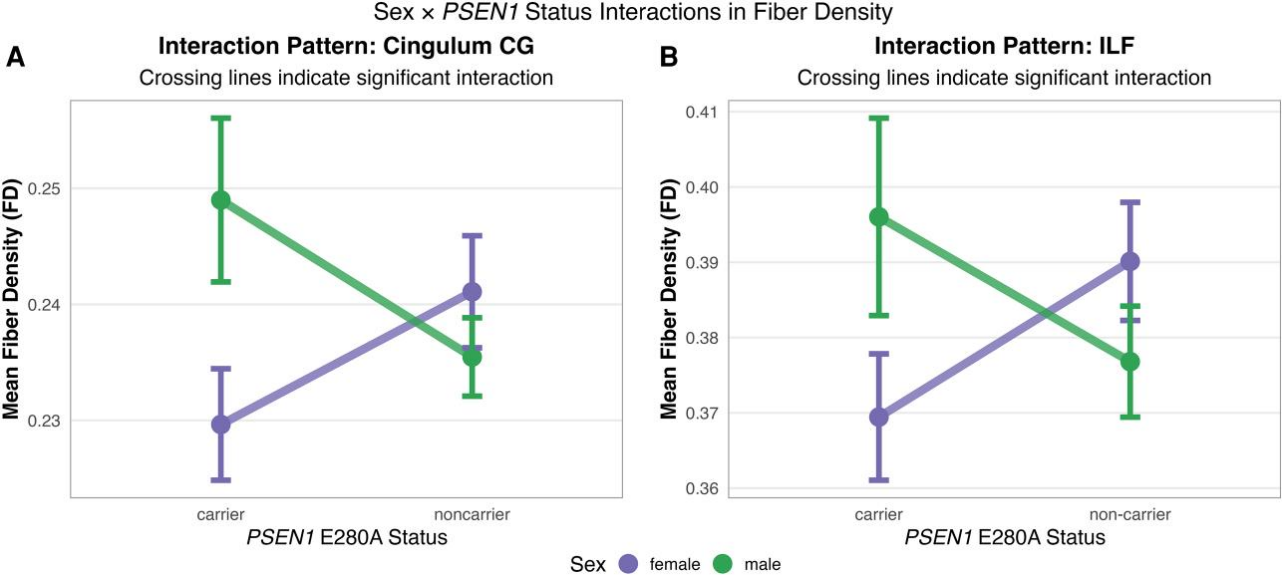
**Sex × *PSEN1* E280A carrier status interactions for white matter fibre density.** Interaction plots showing sex-specific differences in white matter integrity between *PSEN1* E280A mutation carriers (female *n* = 18, male *n* = 12) and non-carriers (female *n* = 18, male *n* = 15), analysed using linear regression with sex × carrier status interaction terms, controlling for age and intracranial volume. **(A)** Right cingulum (cingulate gyrus portion) fibre density demonstrates significant sex × carrier status interaction (β = −0.02, *t* = −2.38, *P* = 0.021), with male carriers showing higher fibre density than female carriers, while this pattern reverses in non-carriers. **(B)** Right inferior longitudinal fasciculus fibre density shows similar sex × carrier status interaction (β = −0.04, *t* = −2.14, *P* = 0.037), with male carriers exhibiting greater fibre density than female carriers. Points represent group means with 95% confidence intervals. Individual data points are overlaid. Each point represents one measurement per participant.

Significant interactions between genetic status and sex were detected in FD measures, indicating that the impact of *PSEN1* E280A mutation status on white matter microstructure differed between males and females. Specifically, interactions were observed in the right cingulum (cingulate gyrus portion), (β = −0.02, 95% CI [−0.04, −0.004], *P* = 0.021) and right inferior longitudinal fasciculus (β = −0.04% CI [−0.07, −0.003], *P* = 0.037), with females showing higher FD values in non-carriers but lower values in carriers compared to the pattern observed in males (Fig. 2). After FDR correction, these interactions did not remain statistically significant.

## Sex-moderated pathology-white matter relations

### Sex differences in amyloid-white matter relations

**Figure 3:**
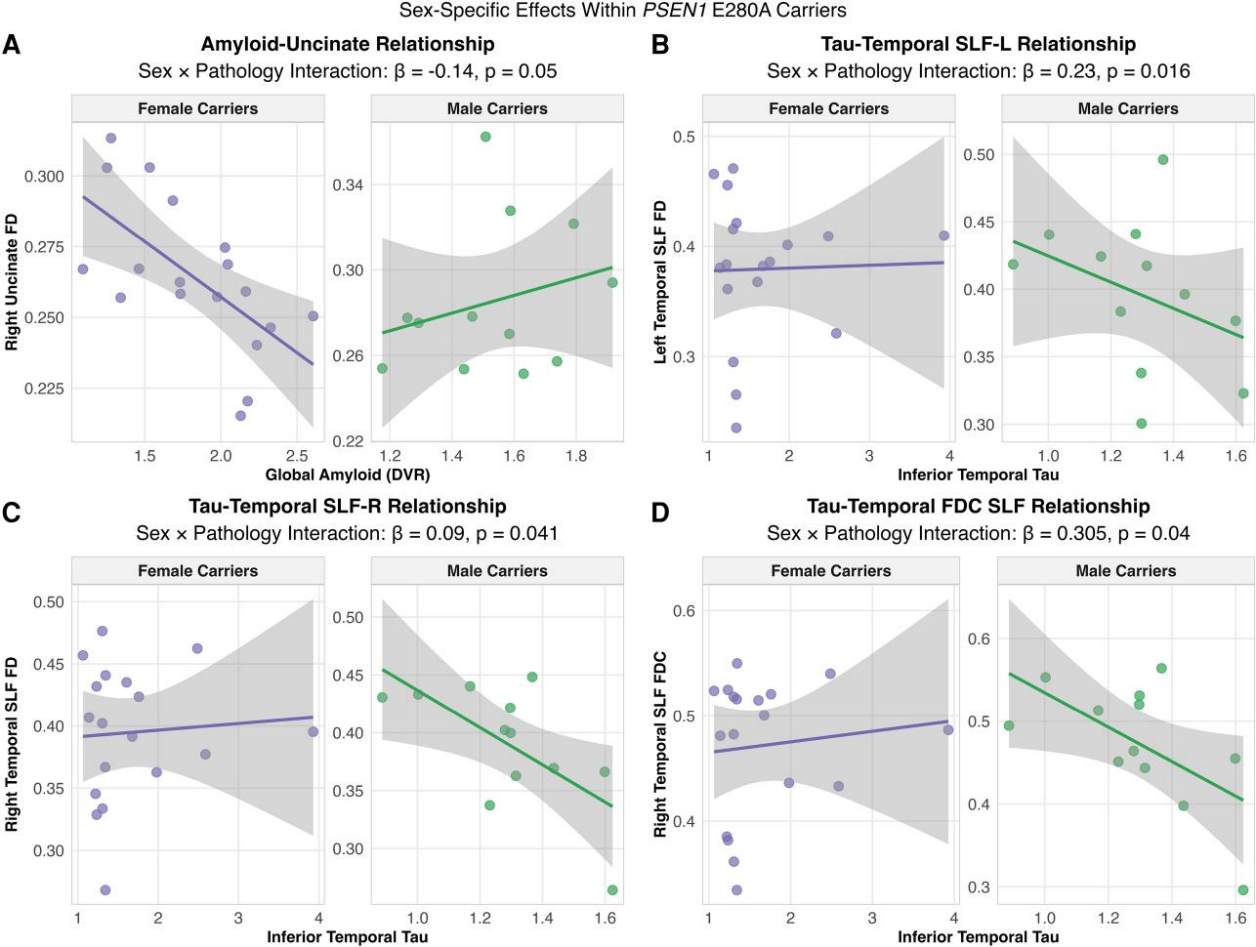
**Sex-specific effects of pathological proteins on white matter integrity within *PSEN1* E280A carriers.** Sex-stratified relationships between pathological protein burden and white matter measures in mutation carriers (female *n* = 18, male *n* = 12), analysed using linear regression with sex × pathology interaction terms, controlling for age and intracranial volume. (**A**) Global amyloid-right uncinate fasciculus fibre density relationship demonstrates significant sex × pathology interaction (β = −0.14, *t* = −2.01, *P* = 0.05), with females showing negative associations while males show positive associations. (B–D) Inferior temporal tau relationships with superior longitudinal fasciculus (SLF) measures show consistent patterns where males exhibit stronger negative associations than females: (**B**) Left temporal SLF fibre density (β = 0.23, *t* = 2.52, *P* = 0.016), (**C**) Right temporal SLF fibre density (β = 0.09, *t* = 2.11, *P* = 0.041) and (**D**) Right temporal SLF FDC (β = 0.305, *t* = 2.13, *P* = 0.04). Points represent individual carriers; lines show sex-specific regression fits with 95% confidence intervals. Individual data points are shown for both sexes. Each point represents one measurement per participant. Findings did not survive false discovery rate correction and should be interpreted as exploratory.

For Aβ pathology, FD in the right uncinate fasciculus showed a trending sex interaction (β = −0.14, 95% CI [−0.24, −0.04], *P* = 0.05), suggesting divergent patterns between males and females. Male carriers exhibited higher FD with greater Aβ burden (β = 0.09), while female carriers showed the decreased FD with greater Aβ burden (β = −0.04) (Fig. 3).

### Sex differences in tau-white matter relations

For inferior temporal tau pathology, multiple temporal superior longitudinal fasciculus measures showed sex interactions (right temporal superior longitudinal fasciculus FD: β = 0.23, 95% CI [0.14, 0.34], *P* = 0.016; right temporal superior longitudinal fasciculus FDC: β = 0.305, 95% CI [0.21, 0.40], *P* = 0.04; left temporal superior longitudinal fasciculus FD: β = 0.09, 95% CI [0.004, 0.19], *P* = 0.04). However, after FDR correction, none of these sex × pathology interactions remained statistically significant (all *P*_FDR > 0.05), indicating that these findings should be interpreted as exploratory and hypothesis-generating rather than definitive evidence of sex-specific vulnerability patterns (Fig. 3). It is important to note that despite female carriers having higher baseline tau levels than male carriers across all regions, females demonstrated more preserved fibre integrity with increasing tau burden compared to males. This pattern indicates that while both sexes show white matter deterioration with increasing tau pathology, the rate of deterioration is slower in females than males. The deleterious effect of tau pathology on the superior longitudinal fasciculus was more pronounced in male carriers than in female carriers.

### Sex-moderated white matter-cognition relations

**Figure 4:**
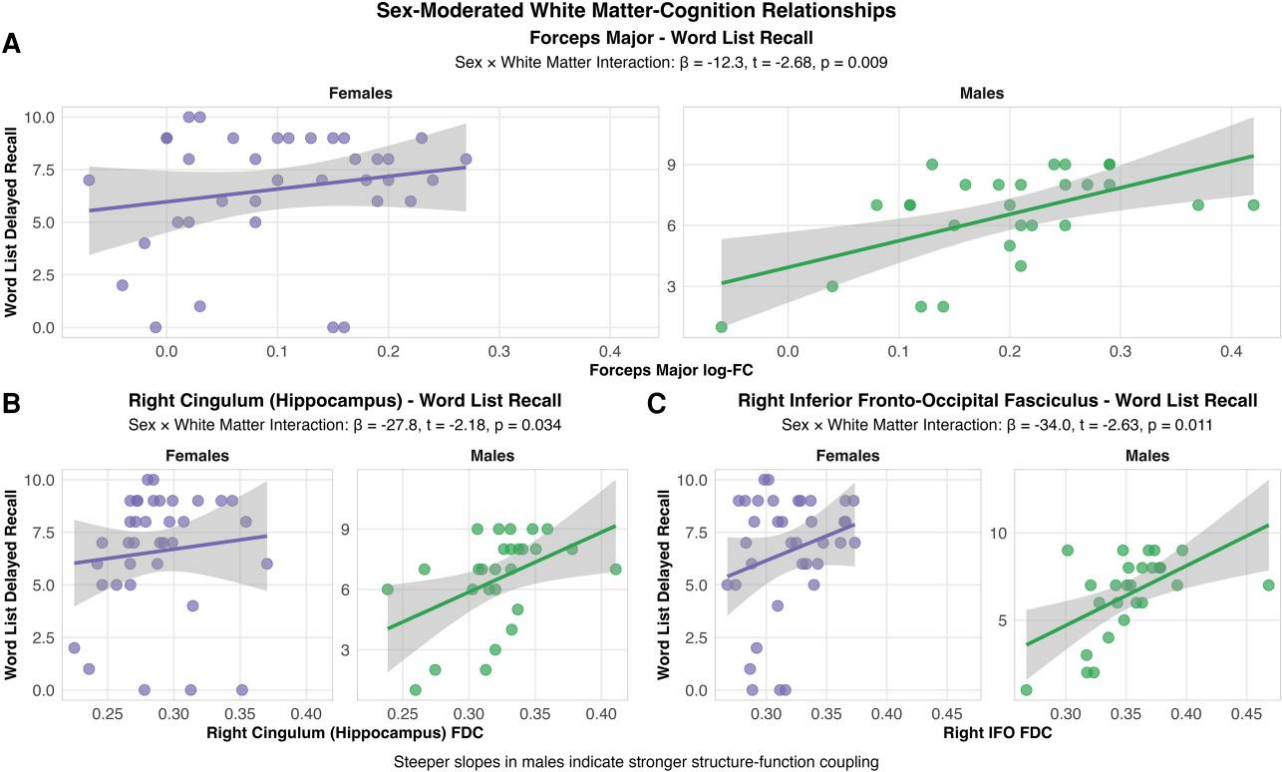
**Sex-moderated white matter–cognition relationships demonstrate male advantage in structure-function coupling.** Scatter plots show representative examples of relationships between white matter integrity and memory performance stratified by sex (males *n* = 27, females *n* = 36), analysed using linear regression with sex × white matter interaction terms, controlling for *PSEN1* E280A status, age, intracranial volume and entorhinal tau burden. All findings shown remained significant after controlling for tau pathology at the uncorrected level**. (A)** Forceps major fibre cross-section (log-FC) and CERAD word list delayed recall show significant sex × log-FC interaction (β = −12.3, *t* = −2.68, *P* = 0.009). **(B)** Right cingulum (hippocampus portion) FDC and CERAD word list delayed recall show significant sex × FDC interaction (β = −27.8, *t* = −2.18, *P* = 0.034). **(C)** Right inferior fronto-occipital fasciculus FDC and CERAD word list delayed recall show significant sex × FDC interaction (β = −34.0, *t* = −2.63, *P* = 0.011). Additional significant interactions (combined inferior fronto-occipital fasciculus and right anterior thalamic radiation) are reported in the Results section. All interactions demonstrate stronger structure–function coupling in males than females, as shown by steeper regression slopes. Points represent individual participants; lines show sex-specific regression fits with 95% confidence intervals.

**Figure 5:**
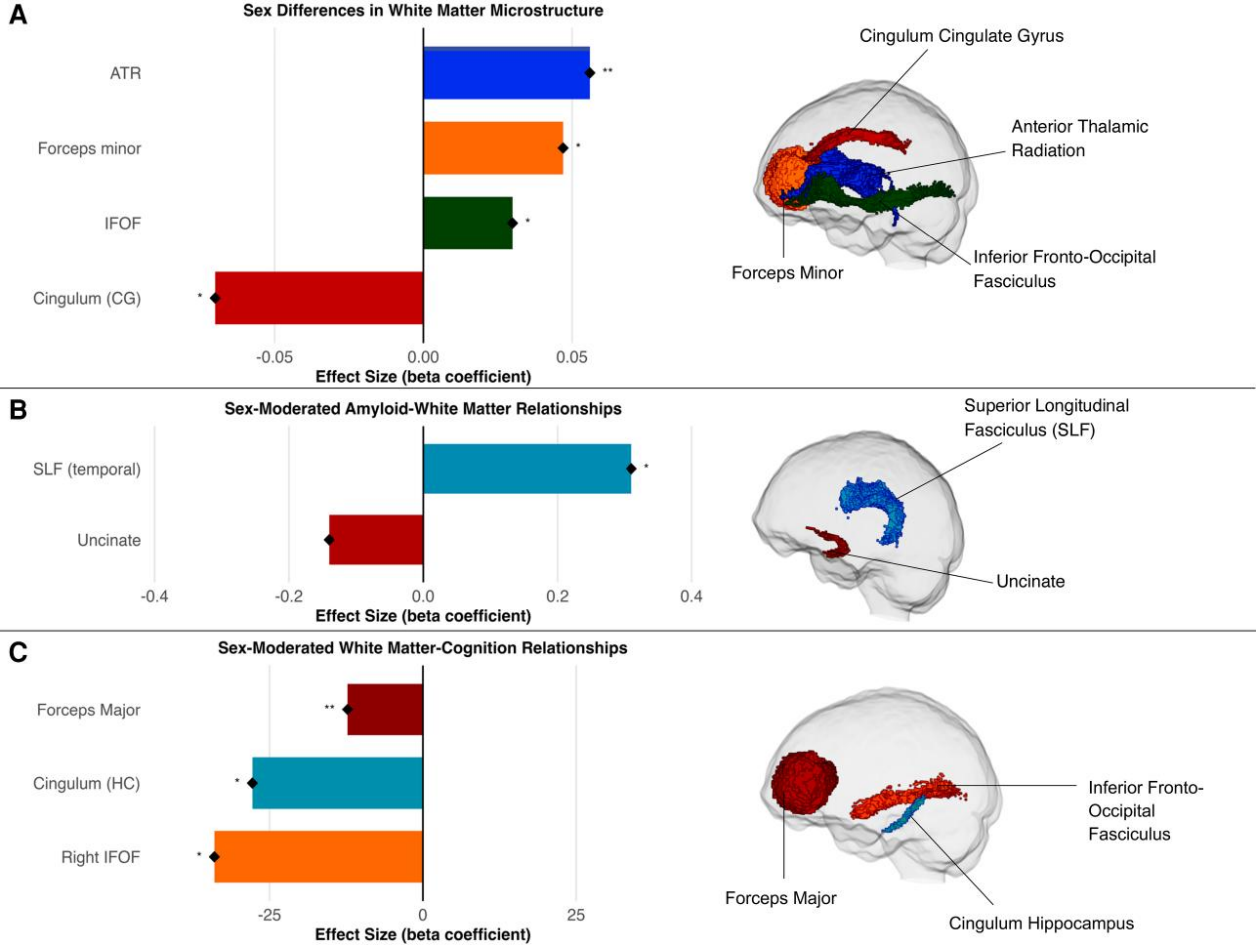
**Sex differences and sex-moderated relationships in white matter microstructure among *PSEN1* E280A mutation carriers and non-carriers.** Summary figure showing anatomical locations of significant white matter findings from linear regression analyses (total *n* = 63: female carriers *n* = 18, male carriers *n* = 12, female non-carriers *n* = 18, male non-carriers *n* = 15). (**A**) Sex differences in fibre cross-section (log-FC) controlling for *PSEN1* E280A status, age and intracranial volume. Effect sizes represent standardized beta coefficients, with positive values indicating higher log-FC in females compared to males (female > male). (**B**) Significant sex × pathology interactions among carriers only, controlling for age and intracranial volume. Effect sizes represent the interaction term beta coefficients from models testing whether the relationship between pathology and white matter integrity differs by sex. Positive values indicate that the pathology–white matter relationship is more positive (or less negative) in females than males. (**C**) Sex-moderated white matter–cognition relationships across full sample, controlling for *PSEN1* E280A status, age, intracranial volume and entorhinal tau burden. Effect sizes represent the sex × white matter interaction term beta coefficients, with positive values indicating that males derive greater cognitive benefit from preserved white matter integrity than females (i.e. stronger positive association in males). Visualizations show anatomical locations of significant white matter tracts using the same colour scheme as the bar plots. Significance levels: *P < 0.05, **P < 0.01 (uncorrected for multiple comparisons). Note: Pathology-related findings in panel B are exploratory and did not survive false discovery rate correction; they should be interpreted with caution pending replication in independent samples. ATR = anterior thalamic radiation; CG = cingulate gyrus; IFOF = inferior fronto-occipital fasciculus; HC = hippocampus.

When examining memory performance, we identified significant sex-moderated relations between white matter integrity and cognitive function. We observed significant white matter × sex interactions for fibre cross-section (log-FC) of the forceps major for word list recall (β = −12.8, 95% CI [−20.13, −5.49], *P* = 0.001), FDC of the right cingulum (hippocampus portion) for word list recall (β = −35.0, 95% CI [−58.4, −11.6], *P* = 0.014), FDC of the right and combined inferior fronto-occipital fasciculus for word list recall (right: β = −35.3, 95% CI [−62.8, −7.8], *P* = 0.018; combined: β = −33.2, 95% CI [−58.2, −8.2], *P* = 0.030), and FDC of the right anterior thalamic radiation for word list recall (β = −25.6, 95% CI [−50.8, −0.4], *P* = 0.048) (Fig. 4). To address potential confounding by tau pathology, we repeated analyses controlling for entorhinal tau burden; all five sex × white matter interactions remained significant (forceps major: β = −12.3, *P* = 0.009; right cingulum: β = −27.8, *P* = 0.034; right inferior fronto-occipital fasciculus: β = −34.0, *P* = 0.011; combined inferior fronto-occipital fasciculus: β = −31.2, *P* = 0.023; right anterior thalamic radiation: β = −25.5, *P* = 0.024), though findings did not survive false discovery rate correction (Fig. 5). These results provide exploratory evidence that sex-specific structure–function coupling patterns persist independent of tau pathology burden. All interactions demonstrated male advantage in structure–function coupling.

## Discussion

White matter degeneration is a common structural alteration in individuals with Ad that contributes significantly to neurodegeneration, with emerging evidence suggesting sex disparities in white matter abnormalities in Ad. To address the limited research on sex differences in white matter microstructure across the Ad continuum, we leveraged a unique cohort of *PSEN1* E280A mutation carriers to characterize sex-specific patterns of white matter integrity in preclinical ADAD, focusing on associations with known biomarkers of Ad progression, cognition and their potential moderation by sex.

Exploratory analyses revealed sex-related differences in multiple pathways, with females showing higher fibre cross-section in the anterior thalamic radiation, forceps minor and inferior fronto-occipital fasciculus. This indicates that females have larger, more preserved white matter tract diameter, reflecting better structural integrity in these areas than males. In the context of Ad, this suggest that females may have preserved white matter structure in these tracts compared to males. These findings are consistent with previous reports demonstrating that males exhibit greater white matter neurodegeneration in Ad, including higher free-water indices and more pronounced microstructural damage.^12^ Our identified tracts align with previously reported sex-differential regions, particularly the anterior thalamic radiation, strengthening evidence for sex-specific protective mechanisms in white matter integrity during Ad progression and highlighting the importance of considering sex as a biological variable in Ad research.^9,19,20^

Exploratory analyses of the relations between amyloid and tau-PET and markers of white matter degeneration revealed potential sex-specific patterns of vulnerability and resilience. Although none of these interactions survived correction for multiple comparisons, the observed patterns suggest potential sex differences in white matter vulnerability to pathology that warrant investigation in larger cohorts. Notably, while fibre integrity declined with increasing tau burden in both sexes, female carriers showed a slower rate of decline in the temporal superior longitudinal fasciculus compared to males suggesting potential sex-specific protective mechanisms. The superior longitudinal fasciculus, critical for language processing and working memory, plays an essential role in cognitive reserve mechanisms that may help maintain function despite advancing pathology. Female carriers’ relative preservation of this tract may contribute to their frequently reported advantage in verbal memory tasks. By contrast, the right uncinate fasciculus showed greater vulnerability in females than males with increasing amyloid burden. This highlights the complex, tract-specific nature of sex differences in white matter degeneration, where females may show both protective and vulnerability patterns across different memory-related circuits.^38-42^ This differential vulnerability across memory-related tracts suggests that tau pathology may affect white matter circuits differently in males and females.^38,39^ The spatial correspondence between regional pathology accumulation and white matter tract degeneration in connected pathways, observed even after controlling for age, suggests that Ad protein aggregation directly or indirectly impacts fibre pathways in sex-specific ways. This integration of structural and molecular signals reveals that white matter vulnerability tracks differently with tau versus amyloid pathology, and that these relationships diverge by sex. Such pathology–structure coupling may help explain why males and females exhibit different clinical trajectories despite similar overall pathological burden. These tract-specific differences may reflect underlying sex differences in white matter susceptibility to neurodegeneration.^43^

The relation between white matter integrity and cognitive performance revealed distinct sex-specific patterns that highlight distinct protective mechanisms. Male carriers demonstrated stronger structure–function coupling, showing greater cognitive benefit from preserved white matter microstructure across multiple tracts including the cingulum portion of the hippocampus, forceps major and corticospinal tract. Male carriers demonstrated stronger structure–function coupling, deriving greater cognitive benefit from preserved white matter microstructure across multiple tracts including the cingulum portion of the hippocampus, forceps major and corticospinal tract.

In this study, we examined sex differences in white matter integrity in ADAD using biologically meaningful measures reflecting fibre tract morphology and microstructure. A key strength is our unique *PSEN1* E280A cohort, which allows examination of white matter alterations without ageing confounds, critical as ageing alone can produce white matter changes unrelated to dementia.

The study also had several limitations. While our single-mutation kindred provides methodological advantages, the sample size is very limited (*n* = 63), constraining statistical power and necessitating cautious interpretation of findings that did not survive correction for multiple comparisons. Although considerable overlap exists between autosomal dominant Ad and sporadic Ad pathology, the extent of generalizability to sporadic Ad remains uncertain and our findings should be replicated in sporadic Ad cohorts.

From a methodological standpoint, several considerations should be noted. The significant age differences across sex × mutation groups (*P* = 0.007) represent a potential confound, as age-related white matter changes could interact with disease-related changes in complex ways. Although all models included age as a covariate, statistical adjustment may not fully capture non-linear or multiplicative age effects, particularly given the wide age range and correlation between age and pathological burden in mutation carriers. Additionally, our diffusion MRI acquisition parameters, including 30 gradient directions and a b-value of 700 s/mm^2^, may limit the interpretability of FD estimates. While the use of SS3T-CSD helps mitigate some limitations associated with single-shell, low b-value acquisitions, lower b-values provide reduced sensitivity to the intra-axonal compartment and less suppression of extra-axonal signal, which can compromise the precision of FD quantification. Higher b-values and more extensive angular sampling would provide more reliable quantification of fibre-specific microstructure.

Finally, the cross-sectional design limits causal inferences about relations between pathology, white matter integrity and cognition, though in this cohort age serves as a proxy for disease progression given the predictable onset of autosomal dominant Ad. Longitudinal studies are needed to clarify the progression of white matter degeneration in relation to clinical and cognitive outcomes in both females and males.

## Conclusions

In sum, our findings reveal exploratory sex-specific patterns of white matter microstructural alterations in ADAD cohort, with females showing preserved fibre cross-section in key tracts including the anterior thalamic radiation, forceps minor and inferior fronto-occipital fasciculus, while showing slower rates of white matter deterioration with increasing tau pathology in memory-related circuits such as the superior longitudinal fasciculus. Males demonstrated advantages in structure–function coupling, deriving greater cognitive benefit from preserved white matter integrity. The maintenance of structure–function relations in females in the presence of Ad pathology suggests potential sex-specific protective mechanisms that may inform targeted therapeutic approaches. Together, these data provide insights into the pathophysiology of Ad and suggest the potential utility of considering sex differences in the development of personalized interventions and clinical trials for persons with Ad.

## Supplementary Material

fcag081_Supplementary_Data

## Data Availability

Anonymized clinical, genetic and imaging data are available upon request, subject to an internal review by Y.T.Q. to ensure that the participants’ anonymity, confidentiality and *PSEN1* E280A carrier or non-carrier status are protected. Data requests will be considered based on a proposal review, and completion of a data sharing agreement, in accordance with the University of Antioquia and Mass General Brigham institutional guidelines. Please submit data requests to Y.T.Q. Code for this project is available at the following link: https://github.com/averi-giudicessi/colbos_dwi_scripts.git.
