# Supplementary material for: Sex differences in diffusion-weighted imaging outcomes in autosomal dominant Alzheimer’s disease
Source: Brain Commun. 2026 Mar 12;8(2):fcag081. doi: 10.1093/braincomms/fcag081 (PMC13012221; doi:10.1093/braincomms/fcag081)
Supplement: fcag081_Supplementary_Data [file fcag081_supplementary_data.docx]

**Supplementary Table S1**: Outlier Assessment for White Matter Microstructure and AD Pathology Measures

| **Category** | **Measure** | **N** | **Mean** | **SD** | **Lower Threshold (Mean - 3SD)** | **Upper Threshold (Mean + 3SD)** | **N Outliers** | **Outlier Value** |
| --- | --- | --- | --- | --- | --- | --- | --- | --- |
| Fiber Density (FD) | Anterior Thalamic Radiation (L) | 63 | 0.311 | 0.031 | 0.217 | 0.405 | 0 | None |
|  | Anterior Thalamic Radiation (R) | 63 | 0.300 | 0.032 | 0.204 | 0.397 | 0 | None |
|  | Corticospinal Tract (L) | 63 | 0.457 | 0.036 | 0.348 | 0.566 | 2 | 0.341, 0.575 |
|  | Corticospinal Tract (R) | 63 | 0.464 | 0.034 | 0.363 | 0.564 | 0 | None |
|  | Cingulum Cingulate Gyrus (L) | 63 | 0.243 | 0.021 | 0.180 | 0.306 | 0 | None |
|  | Cingulum Cingulate Gyrus (R) | 63 | 0.238 | 0.021 | 0.176 | 0.300 | 0 | None |
|  | Cingulum Hippocampus (L) | 63 | 0.318 | 0.034 | 0.217 | 0.420 | 0 | None |
|  | Cingulum Hippocampus (R) | 63 | 0.298 | 0.030 | 0.209 | 0.386 | 0 | None |
|  | Forceps Major | 63 | 0.359 | 0.029 | 0.272 | 0.447 | 0 | None |
|  | Forceps Minor | 63 | 0.307 | 0.024 | 0.234 | 0.379 | 0 | None |
|  | IFO (L) | 63 | 0.304 | 0.027 | 0.223 | 0.385 | 1 | 0.4 |
|  | IFO (R) | 63 | 0.309 | 0.026 | 0.232 | 0.387 | 1 | 0.4 |
|  | ILF (L) | 63 | 0.371 | 0.033 | 0.272 | 0.469 | 0 | None |
|  | ILF (R) | 63 | 0.382 | 0.036 | 0.274 | 0.491 | 1 | 0.509 |
|  | SLF (L) | 63 | 0.317 | 0.024 | 0.245 | 0.390 | 1 | 0.391 |
|  | SLF (R) | 63 | 0.315 | 0.024 | 0.243 | 0.387 | 0 | None |
|  | UNC (L) | 63 | 0.282 | 0.031 | 0.190 | 0.374 | 0 | None |
|  | UNC (R) | 63 | 0.276 | 0.029 | 0.188 | 0.364 | 0 | None |
|  | SLF Temporal (L) | 63 | 0.380 | 0.063 | 0.190 | 0.569 | 0 | None |
|  | SLF Temporal (R) | 63 | 0.392 | 0.050 | 0.243 | 0.542 | 0 | None |
| Fiber Density & Cross-section (FDC) | Anterior Thalamic Radiation (L) | 63 | 0.314 | 0.037 | 0.205 | 0.424 | 0 | None |
|  | Anterior Thalamic Radiation (R) | 63 | 0.309 | 0.042 | 0.181 | 0.436 | 0 | None |
|  | Corticospinal Tract (L) | 63 | 0.491 | 0.075 | 0.266 | 0.715 | 1 | 0.734 |
|  | Corticospinal Tract (R) | 63 | 0.500 | 0.081 | 0.256 | 0.745 | 0 | None |
|  | Cingulum Cingulate Gyrus (L) | 63 | 0.255 | 0.047 | 0.114 | 0.396 | 0 | None |
|  | Cingulum Cingulate Gyrus (R) | 63 | 0.247 | 0.044 | 0.116 | 0.377 | 0 | None |
|  | Cingulum Hippocampus (L) | 63 | 0.329 | 0.045 | 0.193 | 0.464 | 1 | 0.483 |
|  | Cingulum Hippocampus (R) | 63 | 0.302 | 0.038 | 0.187 | 0.417 | 0 | None |
|  | Forceps Major | 63 | 0.418 | 0.060 | 0.238 | 0.599 | 1 | 0.649 |
|  | Forceps Minor | 63 | 0.347 | 0.042 | 0.220 | 0.474 | 0 | None |
|  | IFO (L) | 63 | 0.327 | 0.038 | 0.212 | 0.442 | 1 | 0.472 |
|  | IFO (R) | 63 | 0.333 | 0.037 | 0.221 | 0.446 | 1 | 0.468 |
|  | ILF (L) | 63 | 0.418 | 0.052 | 0.262 | 0.573 | 1 | 0.591 |
|  | ILF (R) | 63 | 0.437 | 0.060 | 0.256 | 0.618 | 1 | 0.675 |
|  | SLF (L) | 63 | 0.350 | 0.044 | 0.219 | 0.481 | 0 | None |
|  | SLF (R) | 63 | 0.350 | 0.049 | 0.204 | 0.496 | 0 | None |
|  | UNC (L) | 63 | 0.311 | 0.043 | 0.183 | 0.439 | 0 | None |
|  | UNC (R) | 63 | 0.302 | 0.042 | 0.177 | 0.428 | 0 | None |
|  | SLF Temporal (L) | 63 | 0.456 | 0.084 | 0.203 | 0.708 | 0 | None |
|  | SLF Temporal (R) | 63 | 0.480 | 0.066 | 0.282 | 0.679 | 0 | None |
| Fiber Cross-section (log-FC) | Anterior Thalamic Radiation (L) | 63 | 0.002 | 0.094 | -0.279 | 0.284 | 0 | None |
|  | Anterior Thalamic Radiation (R) | 63 | 0.014 | 0.089 | -0.253 | 0.280 | 0 | None |
|  | Corticospinal Tract (L) | 63 | 0.065 | 0.097 | -0.225 | 0.355 | 0 | None |
|  | Corticospinal Tract (R) | 63 | 0.069 | 0.111 | -0.265 | 0.403 | 0 | None |
|  | Cingulum Cingulate Gyrus (L) | 63 | 0.026 | 0.123 | -0.343 | 0.394 | 0 | None |
|  | Cingulum Cingulate Gyrus (R) | 63 | 0.017 | 0.117 | -0.334 | 0.368 | 0 | None |
|  | Cingulum Hippocampus (L) | 63 | 0.025 | 0.077 | -0.206 | 0.256 | 0 | None |
|  | Cingulum Hippocampus (R) | 63 | 0.009 | 0.087 | -0.253 | 0.270 | 0 | None |
|  | Forceps Major | 63 | 0.142 | 0.105 | -0.173 | 0.457 | 0 | None |
|  | Forceps Minor | 63 | 0.116 | 0.092 | -0.160 | 0.393 | 0 | None |
|  | IFO (L) | 63 | 0.060 | 0.078 | -0.175 | 0.295 | 0 | None |
|  | IFO (R) | 63 | 0.060 | 0.075 | -0.166 | 0.286 | 0 | None |
|  | ILF (L) | 63 | 0.108 | 0.086 | -0.151 | 0.368 | 0 | None |
|  | ILF (R) | 63 | 0.122 | 0.086 | -0.135 | 0.380 | 0 | None |
|  | SLF (L) | 63 | 0.084 | 0.081 | -0.160 | 0.328 | 0 | None |
|  | SLF (R) | 63 | 0.090 | 0.093 | -0.189 | 0.369 | 0 | None |
|  | UNC (L) | 63 | 0.085 | 0.071 | -0.130 | 0.299 | 0 | None |
|  | UNC (R) | 63 | 0.079 | 0.072 | -0.138 | 0.297 | 0 | None |
|  | SLF Temporal (L) | 63 | 0.180 | 0.082 | -0.067 | 0.427 | 0 | None |
|  | SLF Temporal (R) | 63 | 0.198 | 0.078 | -0.036 | 0.431 | 0 | None |
| AD Pathology | Entorhinal Tau SUVR | 63 | 1.383 | 0.603 | -0.426 | 3.192 | 1 | 3.554 |
|  | Inferior Temporal Tau SUVR | 63 | 1.338 | 0.445 | 0.002 | 2.674 | 1 | 3.928 |
|  | Precuneus Tau SUVR | 63 | 1.445 | 0.934 | -1.358 | 4.248 | 2 | 6.242, 5.131 |
|  | Global Amyloid DVR | 63 | 1.397 | 0.398 | 0.203 | 2.591 | 1 | 2.606 |

**Note:** Outliers defined as values >3 standard deviations from the mean across 64 white matter and pathology measures. 18 outlier values were detected across 5 participants (7.9% of sample), all PSEN1 E280A carriers. One outlier was detected in white matter microstructure (right inferior longitudinal fasciculus FD, male carrier), and three in pathology measures within a single female carrier (entorhinal tau SUVR, inferior temporal tau SUVR, and global amyloid DVR). No participants were excluded, as variability reflects clinically meaningful heterogeneity across disease stages in this rare autosomal dominant Alzheimer's disease cohort. Robust regression with MM-estimation was employed in all statistical models to minimize undue influence while preserving complete data.
